# Natural Pregnancy Following Oocyte Retrieval in a Luteal-Phase Progestin-Primed Ovarian Stimulation (PPOS) Protocol With Concurrent Blastocyst Cryopreservation: A Case Report

**DOI:** 10.7759/cureus.90293

**Published:** 2025-08-17

**Authors:** Feras Sendy, Naameh Moussaoumay, Robert Hemmings

**Affiliations:** 1 Obstetrics and Gynecology, King Fahad Medical City, Riyadh, SAU; 2 Reproductive Endocrinology and Infertility, University of Montreal Health Centre (CHUM), Montreal, CAN; 3 Reproductive Endocrinology and Infertility, Ovo Fertility Clinic, Montreal, CAN

**Keywords:** blastocyst, corpus luteum, oocyte retrieval, pregnancy, progesterone-primed ovarian stimulation (ppos) protocols

## Abstract

Ovarian stimulation is used in assisted reproductive technology to help infertile couples achieve pregnancy. Luteal-phase stimulation with progestin-primed ovarian stimulation (PPOS) is a practical approach, as it involves oral medication with fewer daily injections compared to gonadotropin-releasing hormone (GnRH) agonist or GnRH antagonist protocols. Unintended pregnancy during ovarian stimulation or after oocyte pick-up (OPU) is rare but possible. We present the case of a 38-year-old patient with secondary infertility, known to have endometriosis, chronic inflammatory demyelinating polyneuropathy, and low ovarian reserve. She initially underwent ovarian stimulation with an antagonist protocol, which was later switched to luteal-phase PPOS to improve synchronization. For ovulation induction to initiate luteal-phase stimulation, the patient received human chorionic gonadotropin (hCG). A pregnancy test performed during ovarian stimulation yielded a result consistent with exogenous hCG administration. On the day of OPU, a corpus luteum was observed in the right ovary, and two follicles were present in the left ovary. OPU resulted in the retrieval of one oocyte, which developed into a blastocyst and was subsequently cryopreserved. A natural pregnancy was detected by hCG testing 10 days after OPU, prompted by a delayed menstrual cycle and the corpus luteum observed during OPU. Transvaginal ultrasound confirmed an intrauterine pregnancy with a positive fetal heartbeat. This case highlights the importance of considering the possibility of pregnancy even after luteal-phase PPOS and OPU, taking into account both the patient’s clinical context and laboratory findings.

## Introduction

Ovarian stimulation helps recruit multiple follicles for oocyte pick-up (OPU), leading to the production of mature oocytes for in vitro fertilization [1]. Without effective pituitary suppression, premature ovulation may occur in more than 20% of ovarian stimulation cycles [2]. Various stimulation protocols have been developed for pituitary suppression, including gonadotropin-releasing hormone (GnRH) agonists, GnRH antagonists, and progestins [3]. Progestin-primed ovarian stimulation (PPOS) is an effective oral alternative for preventing premature luteinizing hormone (LH) surges during ovarian stimulation [4]. It can be applied in patients with endometriosis, for fertility preservation, and in oocyte donation while maintaining contraception [5-7].

PPOS can be initiated in different ways, including conventional, flexible, random-start, or DuoStim (follicular and luteal phase) approaches [8-10]. Although rare, the risk of an undetected spontaneous pregnancy during luteal-phase stimulation remains possible [11]. Here, we report a case of spontaneous pregnancy after OPU with luteal-phase PPOS, which resulted in the development of a day-6 blastocyst that was subsequently vitrified. Follow-up ultrasound scans after OPU confirmed a viable intrauterine pregnancy.

## Case presentation

A 38-year-old gravida 1 para 0 with a previous miscarriage at seven weeks of gestation after a spontaneous pregnancy presented to Ovo Fertility Center with a three-year history of secondary infertility due to combined factors, including low ovarian reserve and asthenoteratozoospermia. Her medical history was remarkable for endometriosis and chronic inflammatory demyelinating polyneuropathy, with a previous episode of paralysis lasting five months that required hospitalization in the intensive care unit. Her surgical history included tracheostomy, gastrostomy, hysteroscopic polypectomy, and conization of the cervix. She had a body mass index of 18 kg/m², a regular menstrual cycle, and a low ovarian reserve, as indicated by an anti-Müllerian hormone level of 0.51 ng/mL and an antral follicle count of 6.

Her partner, a 38-year-old male, was diagnosed with asthenoteratozoospermia. Semen analysis revealed a sperm concentration of 33 million/mL, progressive motility of 27%, and normal morphology of 3%. His DNA fragmentation index was normal (<5%). Ovarian stimulation with intracytoplasmic sperm injection (ICSI) was recommended.

The patient initially started testosterone gel 1% (Androgel) for three months to improve ovarian stimulation response, followed by stimulation using an antagonist protocol with daily Menopur 300 IU and Rekovelle 12 mcg. Cetrorelix (Cetrotide 250 mcg) was administered for the suppression of premature ovulation. Ultrasound on day 13 of stimulation showed two follicles on the right ovary (21 mm and 15 mm) and four follicles on the left ovary (three of 10 mm and one of 8 mm). Estradiol and progesterone levels on that day were 1,976 pmol/L and 1.74 nmol/L, respectively.

The options discussed with the couple were ovulation induction followed by oocyte retrieval, with the risk of failing to obtain oocytes given the number of visible follicles; ovulation induction with human chorionic gonadotropin (hCG) 5,000 IU, followed by restarting ovarian stimulation five days later with PPOS; or canceling the cycle entirely and considering alternative options. The couple opted for luteal-phase PPOS.

Stimulation was restarted five days after ovulation induction with hCG 5,000 IU using daily Menopur 450 IU, Rekovelle 16 mcg, and Provera 10 mg. Ultrasound on day 6 of stimulation showed three follicles on the right ovary (24 mm, 6 mm, and 4 mm) and six follicles on the left ovary (24 mm, 20 mm, 16 mm, 20 mm, 12 mm, and 10 mm). Estradiol was 2,521 pmol/L. The patient reported a positive urinary pregnancy test. Serum hCG was measured at 10 IU/L, which was considered residual from the hCG administered 10 days earlier. Dual trigger with hCG 5,000 IU and Suprefact 1 mg was prescribed, and OPU was scheduled 36 hours later.

On the day of OPU, a corpus luteum was observed on the right ovary, and two follicles were seen on the left ovary, along with free fluid in the Douglas pouch. Both follicles were aspirated using a double-lumen needle with follicular flushing, and the free fluid was aspirated at the end of OPU. One mature oocyte was retrieved and used for ICSI. A day-6 blastocyst was obtained and vitrified.

**Figure 1:**
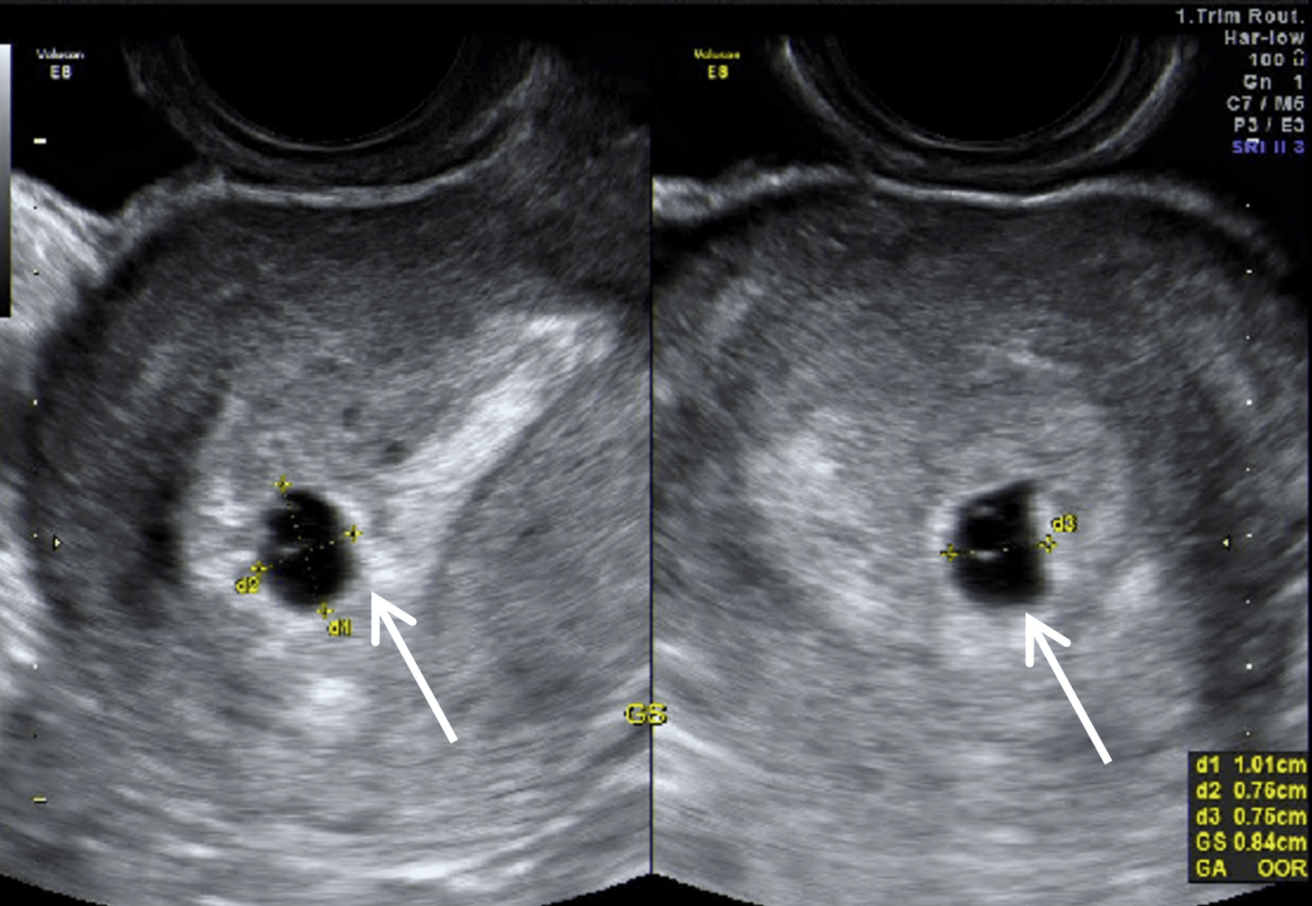
Transvaginal ultrasound 17 days after OPU The white arrow indicates an intrauterine gestational sac with a yolk sac. GS, gestational sac, measured in three dimensions (d1, d2, d3); OPU, oocyte pick-up

Due to delayed menstruation and the presence of a corpus luteum on the day of OPU, a serum pregnancy test was performed 10 days after retrieval, showing hCG of 1,784 IU/L. Forty-eight hours later, hCG increased to 2,991 IU/L. A transvaginal ultrasound performed 17 days after OPU demonstrated an intrauterine gestational sac measuring 0.84 cm with a yolk sac (Figure 1).

**Figure 2:**
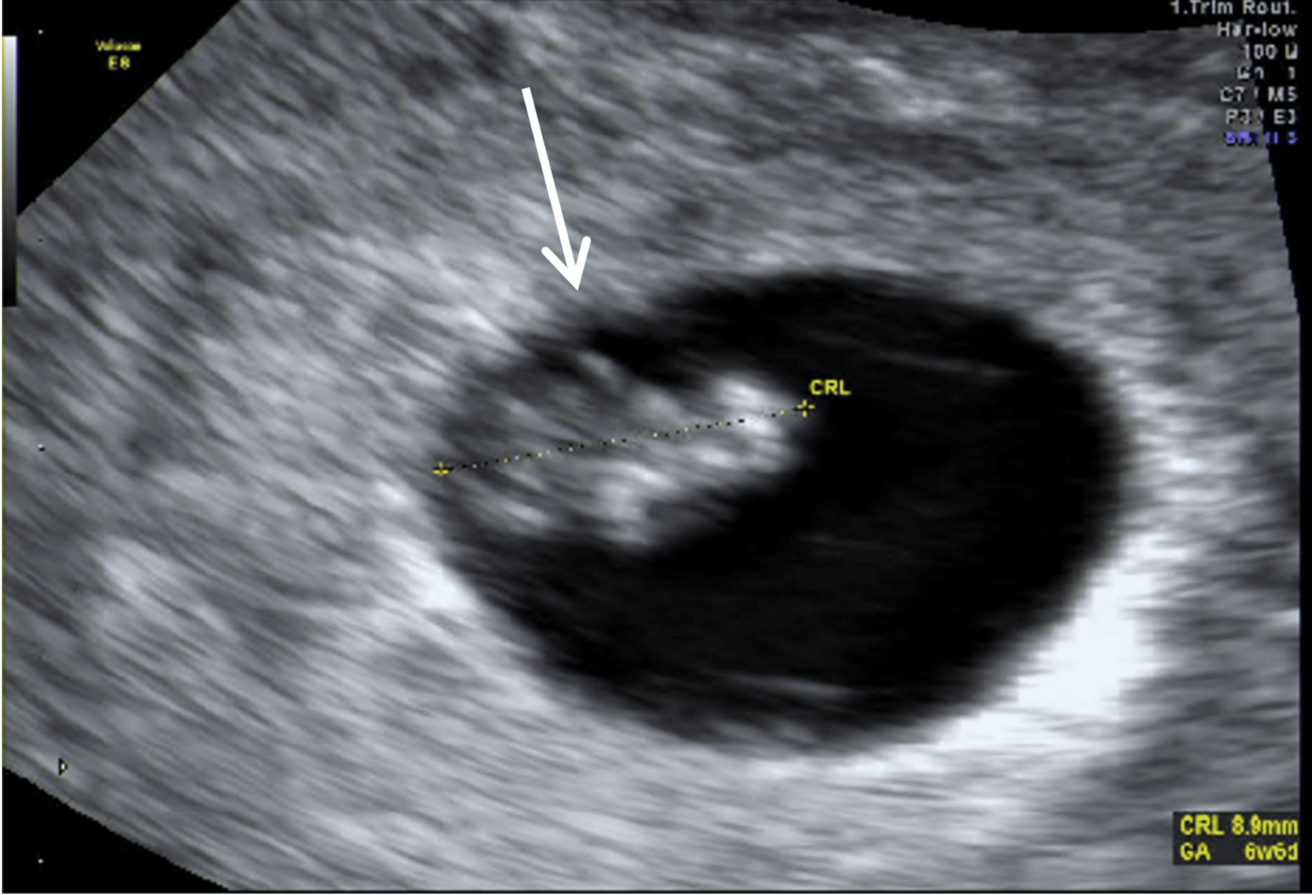
Follow-up transvaginal ultrasound 27 days after OPU The white arrow indicates an intrauterine gestational sac with a CRL corresponding to six weeks and six days. CRL, crown-rump length; GA, gestational age; OPU, oocyte pick-up

She was therefore recommended to start Aspirin 81 mg once daily and vaginal progesterone (Prometrium 100 mg twice daily). A follow-up ultrasound 10 days later revealed an intrauterine gestational sac with a yolk sac, fetal pole, and positive fetal cardiac activity, with a crown-rump length corresponding to six weeks and six days (Figure 2).

**Figure 3:**
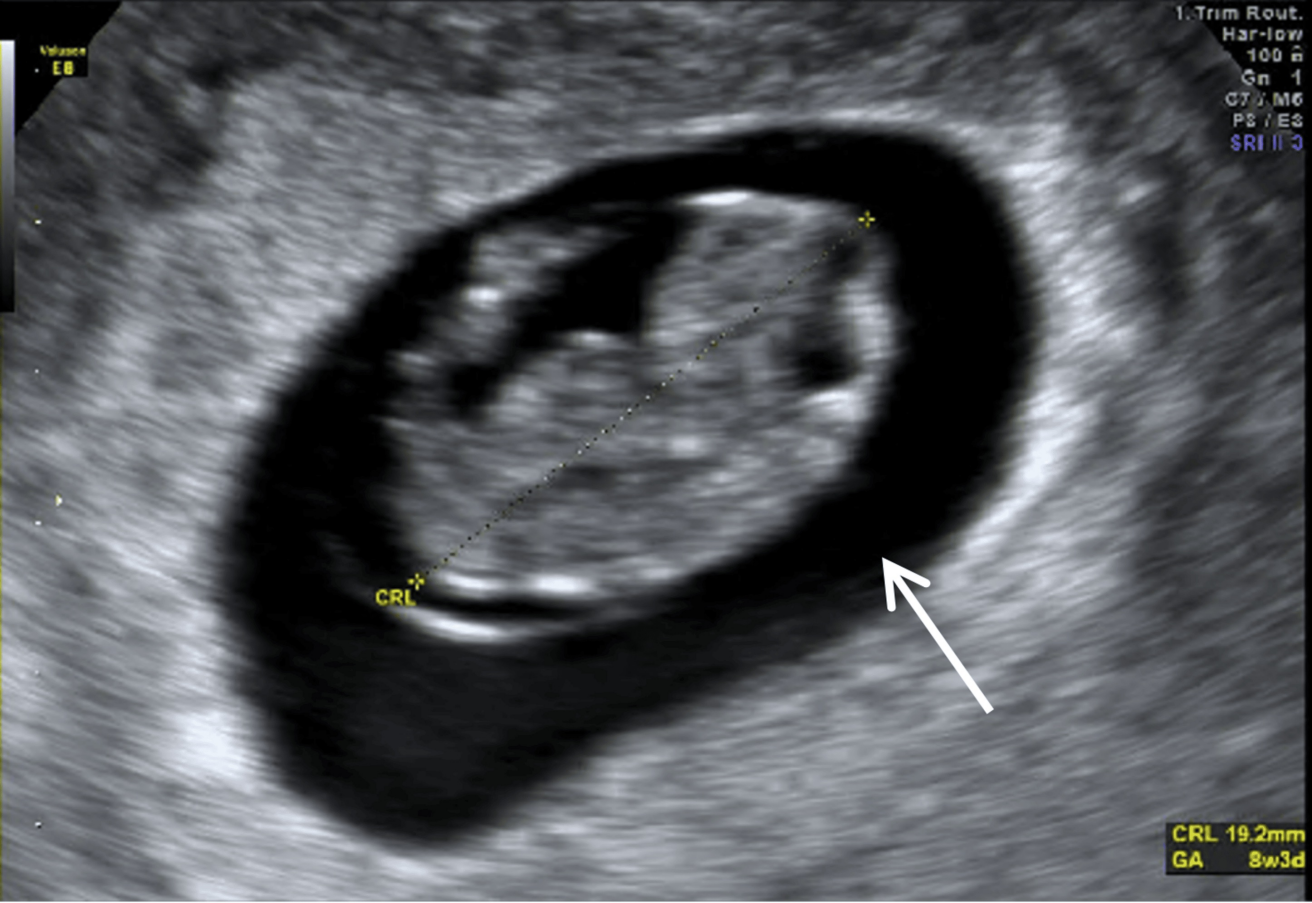
Latest transvaginal ultrasound performed at our clinic, showing adequate fetal growth The white arrow indicates an intrauterine gestational sac with a CRL corresponding to eight weeks and three days. CRL, crown-rump length; GA, gestational age

A subsequent ultrasound demonstrated adequate fetal growth (Figure 3).

All her hCG blood tests were performed in the same laboratory. Written informed consent was obtained from the patient for the use of her medical information and accompanying images in this case report.

## Discussion

This is the first reported case of a viable spontaneous pregnancy after OPU in a luteal-phase PPOS protocol, which also resulted in the development of a day-6 blastocyst that was subsequently vitrified. Luteal-phase PPOS was suggested due to the asynchrony observed during ovarian stimulation with the antagonist protocol. The objective of follicular synchronization was achieved with luteal-phase PPOS, as follicular sizes became more comparable.

For many years, GnRH agonist and GnRH antagonist protocols have been used for ovarian stimulation [12,13]. The mechanism of action of GnRH agonists involves a flare-up effect of follicle-stimulating hormone and LH [13]. However, prolonged exposure leads to receptor downregulation. In contrast, GnRH antagonists competitively block GnRH receptors, resulting in immediate suppression [12]. Progesterone in the luteal phase suppresses endogenous gonadotropins and, being an oral medication, offers an advantage over GnRH agonists or antagonists [4]. Consequently, fewer injections are required, which can be more practical for many patients.

The positive pregnancy test result of 10 IU/L during luteal-phase PPOS, obtained 10 days after ovulation induction with hCG, was considered to reflect residual hCG from the trigger injection. A study evaluating hCG levels in 34 patients undergoing ovarian stimulation with hCG for ovulation induction found that serum hCG remained detectable at 4-14 IU/L 10 days later [14]. This evidence supports the likelihood of exogenous hCG in our case.

During OPU, a corpus luteum was observed in the left ovary along with free fluid in the Douglas pouch, which may have indicated ovulation. Retrieval of only one oocyte during OPU further supports this possibility. However, distinguishing a corpus luteum from a follicle in luteal-phase ovarian stimulation can be challenging [15,16]. In addition, we avoided puncturing the corpus luteum to reduce the risk of bleeding due to its vascularization. Ten days after OPU, the patient’s hCG level was 1,784 IU/L, consistent with pregnancy. Two days later, hCG had risen to 2,991 IU/L. The minimal reported increase in hCG for a viable intrauterine pregnancy over 48 hours is 53% [17]. Our patient showed a 67.65% increase, supporting the diagnosis of a viable intrauterine pregnancy.

The risk of an undetected natural pregnancy during luteal-phase stimulation has been reported as 2.1% across 488 cycles [11]. Within this cohort, only one case of pregnancy after OPU was reported with an antagonist stimulation protocol, though without a detailed description [11]. Moreover, only two biochemical pregnancies have been reported during PPOS [11]. The authors noted that persistently elevated progesterone levels were the key to diagnosing all but one of the reported pregnancies (nine out of 10 cases) [11]. It is arguable that, had progesterone been measured in our case, the diagnosis might have been achieved in a similar manner. At our fertility center, progesterone is not measured on day 6 in PPOS cycles, as premature ovulation is considered rare at this stage; however, it is assessed at the subsequent follicular monitoring ultrasound.

hCG is produced by the embryo approximately seven days post-fertilization [18], with syncytiotrophoblasts contributing to its production after implantation [19]. In our case, the pregnancy test performed before OPU showed low hCG levels consistent with residual hormone from the ovulation trigger, suggesting that conception occurred after retrieval.

The strength of this case is that, to our knowledge, it represents the first reported instance of natural conception occurring after oocyte retrieval in a luteal-phase PPOS protocol, resulting in a viable intrauterine pregnancy alongside the development of a day-6 blastocyst that was successfully vitrified. Serial hCG monitoring facilitated the timely diagnosis of an ongoing pregnancy.

The main limitation of this case is the absence of progesterone measurements, which can serve as an indirect indicator of pregnancy, as highlighted in previous reports. Further research is warranted to strengthen the current evidence, given the limited number of available cases.

## Conclusions

Serum hCG evaluation is a well-established method for diagnosing pregnancy in cases of delayed menstrual cycles. Although the likelihood of pregnancy during ovarian stimulation or after OPU is low, it remains possible. In cases of suspicion, such as the presence of a corpus luteum, a delayed menstrual cycle, or elevated progesterone levels, an hCG blood test is required to confirm the diagnosis.
